# Chlorophyll Fluorescence-Based High-Throughput Phenotyping Reveals Mechanisms and Enables Rapid Screening of Desiccation-Tolerant Wild Tomato Species

**DOI:** 10.3390/plants15091339

**Published:** 2026-04-28

**Authors:** Sushil S. Changan, Pratapsingh S. Khapte, Priti S. Rathod, Sangram B. Chavan, Vijaysinha D. Kakade, Amrut S. Morade, Yogesh P. Khade, S. Gurumurthy, Chetan S. Sonawane, Ajay Kumar Singh, Kotha Sammi Reddy

**Affiliations:** 1 Indian Council of Agricultural Research—National Institute of Abiotic Stress Management, Baramati 413 115, Maharashtra, India; 2 Indian Council of Agricultural Research—Directorate of Onion and Garlic Research, Rajgurunagar 410 505, Maharashtra, India; 3 Indian Council of Agricultural Research—Indian Agricultural Research Institute, Regional Station, Indore 452 001, Madhya Pradesh, India

**Keywords:** abiotic stresses, chlorophyll fluorescence, photosystem II, wild tomato, QY_max

## Abstract

Desiccation tolerance is a critical adaptive trait that enables plants to survive extreme water loss, yet its physiological basis in tomato and its wild relatives remains poorly understood. In this study, chlorophyll *a* fluorescence imaging was used as a reliable tool to evaluate photosystem II (PSII) response to progressive desiccation. The analysis was conducted in cultivated tomato (*Solanum lycopersicum*) and five wild relatives (*Solanum chilense*, *Solanum habrochaites*, *Solanum peruvianum*, *Solanum pimpinellifolium*, and *Solanum pennellii)*. Detached leaves were subjected to controlled desiccation for up to 50 h. During this period, tissue moisture content (TMC), relative water content (RWC), PSII photochemical efficiency [Fv/Fm; maximum quantum yield (QY_max)], minimal fluorescence (F0), maximal fluorescence (Fm), and variable fluorescence (Fv) were monitored to assess changes in photosynthetic performance. Desiccation caused a significant, moisture-dependent decline in PSII efficiency across all species, with QY_max showing a strong linear relationship with RWC (R^2^ = 0.80–0.90). Interspecific variation was evident as *S. chilense*, *S. habrochaites*, *S. peruvianum*, and S. *pimpinellifolium* exhibited rapid PSII impairment, while *S. lycopersicum* showed moderate tolerance. In contrast, *S. pennellii* maintained higher PSII stability, with 50% loss of efficiency occurring only at lower RWC (30–35%). Overall, chlorophyll fluorescence imaging effectively captured functional diversity in desiccation tolerance, highlighting *S. pennellii* as a valuable genetic resource for improving drought resilience in tomato.

## 1. Introduction

Plants are constantly challenged by various abiotic stresses, including drought, salinity, extreme temperatures, and oxidative stress, all of which can severely disrupt their physiological and biochemical processes, leading to reduced growth and productivity [1]. Among these factors, drought represents one of the most widespread and destructive constraints on global crop yield. Tomato (*Solanum lycopersicum* L.) is one of the most widely cultivated vegetable crops worldwide, valued for its nutritional and economic importance, and serves as a model plant for vegetable research. However, its productivity is severely constrained by abiotic stresses, particularly drought, which is becoming more frequent and intense under changing climatic conditions. Drought stress negatively affects tomato growth and yield by reducing photosynthesis, disturbing osmotic balance, impairing nutrient uptake, and enhancing reactive oxygen species (ROS) accumulation [2,3]. To cope with drought, tomato plants activate various morphological (reduced leaf area, deeper rooting), physiological (stomatal regulation, osmolyte accumulation), and biochemical (antioxidant enzyme activity, phytohormonal signaling) mechanisms [4,5].

Desiccation, the extreme loss of cellular water, represents one of the most damaging forms of abiotic stress in plants. Unlike moderate drought stress, desiccation leads to near-complete dehydration of tissues, resulting in membrane destabilization, protein denaturation, and impaired photosynthesis [6]. Plants have evolved adaptive mechanisms, such as deep root systems, waxy cuticles, and antioxidant accumulation, to mitigate drought-induced desiccation [7,8]. In agricultural systems, desiccation is closely linked to prolonged water deficit, a condition that is becoming increasingly frequent and severe under the current scenario of global climate change [9]. Therefore, understanding the complex mechanism of desiccation tolerance is essential for developing climate-resilient crops with improved water-deficit adaptation.

Tomato has many wild relatives, including *S. pennellii*, *S. chilense*, *S. peruvianum*, *S. pimpinellifolium*, etc., which exhibit significant diversity in abiotic-stress tolerance traits [10]. The wild species of tomato may be considered analogous in terms of genetic distance and cross-compatibility. Cultivated tomato and many of its wild relatives are interfertile, allowing introgression and backcrossing to transfer stress-tolerance traits [11,12]. These wild relatives therefore represent a reservoir of abiotic stress-tolerance alleles that could be harnessed via advanced breeding, genomics, and molecular tools to develop cultivated tomato genotypes that better withstand drought, salinity, and other climatic stresses.

Photosynthesis is the fundamental process that converts solar energy into chemical energy, sustaining life by producing food, oxygen, and essential biomolecules. Photosystem I (PSI) and Photosystem II (PSII) are essential protein complexes in the thylakoid membranes of plant chloroplasts, playing crucial roles in photosynthesis [13]. PSII initiates the light-dependent reactions by absorbing light energy, facilitating water splitting to release oxygen and electrons, and producing ATP for cellular energy [14]. PSI further energizes these electrons using additional light to drive the reduction of NADP+ to NADPH, which is vital for the Calvin cycle and carbon fixation in plants. Together, both photosystems enable plants to efficiently capture solar energy, power metabolic processes, and sustain life by producing oxygen and organic molecules [15]. PSII efficiency is a critical factor in photosynthesis, reflecting the ability of Photosystem II to capture light and drive electron transfer for energy production [16]. Chlorophyll fluorescence is the small fraction of absorbed light that chlorophyll molecules re-emit as red to far red light (around 650–800 nm) when they return from an excited state to the ground state during photosynthesis [17]. Chlorophyll fluorescence kinetics provides a sensitive, robust tool to assess quantum yield of PSII, offering rapid insights into plant physiological health and helps in the early detection of environmental stress impacts on photosynthesis [18]. Since PSII is often the primary site of damage or regulation during abiotic stress, fluorescence kinetics offer a comprehensive information of the plant’s stress-response phenotype. This establishes the technique not merely as a measure of photosynthetic capacity, but as a robust, reliable indicator for evaluating plant resilience and acclimation strategies under adverse conditions. Several stresses hamper photosynthesis by disrupting the photochemical apparatus and reducing CO_2_ assimilation due to stomatal closure, increasing susceptibility to photochemical damage at PSII [19,20]. Khapte et al. [21] investigated desiccation tolerance in wild eggplant species using chlorophyll fluorescence imaging, identifying species with high desiccation stress tolerance, which could serve as valuable genetic resources for developing stress-tolerant eggplant cultivars through breeding programs. Sousaraei et al. [22] evaluated tomato landraces for drought tolerance using comprehensive analyses of chlorophyll fluorescence and growth performance parameters to assess physiological resilience under water deficit conditions. Moreover, Yoshiyama et al. [23] reported that wild tomato species exhibit substantial variation in photosynthetic induction, with faster stomatal responses and smaller, denser stomata enabling higher integrated photosynthesis efficiency measured through gas exchange parameters under natural fluctuating light.

Tomato is generally regarded as a stress-sensitive crop and exhibits limited tolerance to a wide range of abiotic constraints; consequently, its growth and yield are severely restricted by adverse environmental factors, particularly drought and salinity [24]. Therefore, introgression of abiotic stress tolerant traits from wild species represents a promising strategy to mitigate the adverse effects of environmental stresses and to enhance water-use efficiency and crop productivity [25,26]. Most of the studies have identified tolerance in wild tomato species primarily relying on invasive and destructive methods like morphological [27] and physio-biochemical [28] traits. Screening of wild tomato germplasm for abiotic stress resilience, particularly drought, has frequently relied on biochemical indicators to identify promising sources of tolerance. Several wild relatives perform better than cultivated tomato under stress, with reports of enhanced drought and salinity tolerance in specific wild species [29]. However, comprehensive information on abiotic stress tolerance across the broader spectrum of tomato wild relatives remains limited.

Despite many studies identified the tomato wild species germplasm for drought tolerance by using conventional screening/phenotyping methods, there is meagre work on employing ChlaF imaging-based techniques. Here, we address this gap by hypothesizing that genetic variation in desiccation tolerance among tomato species is associated with differences in photosystem II efficiency. To test this, we employed chlorophyll fluorescence imaging as a novel technique to assess PSII sensitivity to desiccation, with the objectives of optimizing the fluorescence protocol and identifying wild tomato species exhibiting superior desiccation tolerance.

## 2. Results

### 2.1. Effect of Desiccation on Tissue Moisture Content and ChlaF

The tissue moisture content (TMC) in leaves of cultivated and wild tomato species was significantly influenced under desiccation treatment (Table S1). As the species exhibited variation in TMC at same time points, they were grouped (76–100%, 51–75%, 26–50%, and 1–25%) to better analyze the relationship between TMC and QY_max. We measured minimal fluorescence (F0), maximum fluorescence (Fm), variable fluorescence (Fv), and the maximum quantum yield of PSII (Fv/Fm) under dark adaptation, also known as QY_max. The ChlaF parameters and maximum quantum yield of PSII were strongly affected by TMC. The TMC significantly influenced all measured ChlaF parameters (F0, Fm, Fv and QY_max), with *p* ≤ 0.001 (Table 1). As TMC decreased, F0 showed a significant increase across all species, indicating increased stress or damage to photosystem reaction centers. The highest TMC group (76–100%) had the lowest F0 value (432.68), whereas the lowest TMC group (1–25%) exhibited the highest F0 value (857.03), significantly higher than all other groups. Conversely, Fm and Fv significantly decreased with decreasing TMC. In the 76–100% TMC group, Fm and Fv were 1541.38 and 1099.91, respectively, declining progressively to their lowest values in the 1–25% TMC group (Fm = 1211.94, Fv = 366.41). The substantial reduction in Fv highlights a marked decline in photochemical efficiency under severe dehydration. The maximum quantum yield of PSII, which is an indicator of maximum photosynthetic efficiency, was also significantly and negatively affected due to reduction in TMC. The highest QY_max value (0.72) was observed in the 76–100% TMC group, consistent with healthy photosynthetic tissue. The QY_max showed a progressive decrease across the desiccation treatments and dropped sharply to 0.28 in the 1–25% TMC group.

### 2.2. Temporal Effect of Desiccation on PSII Efficiency

**Figure 1:**
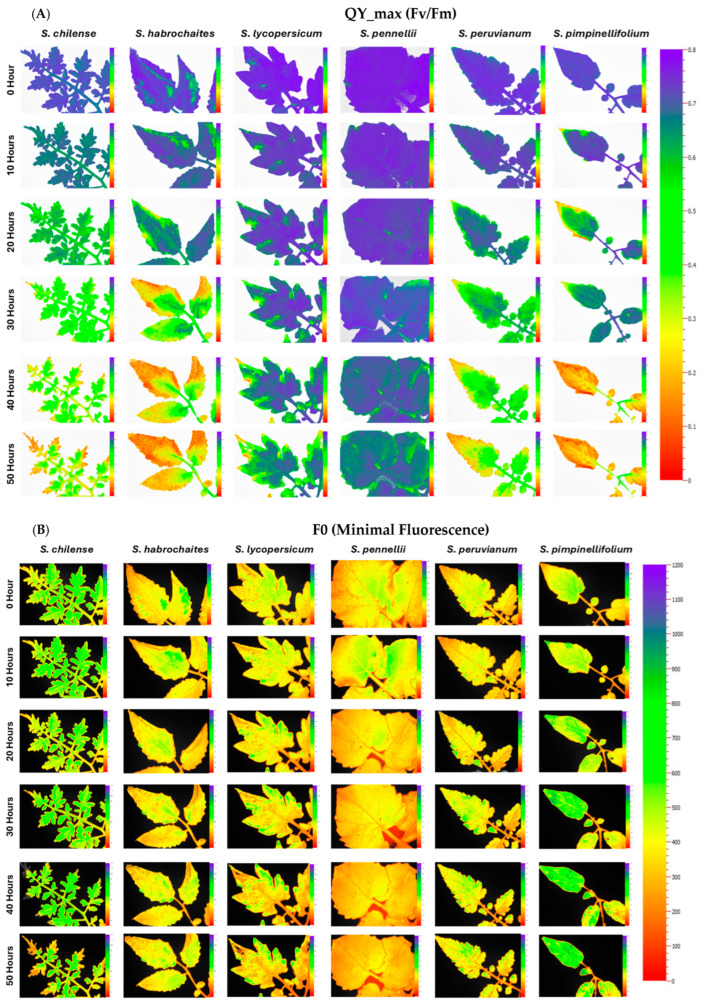

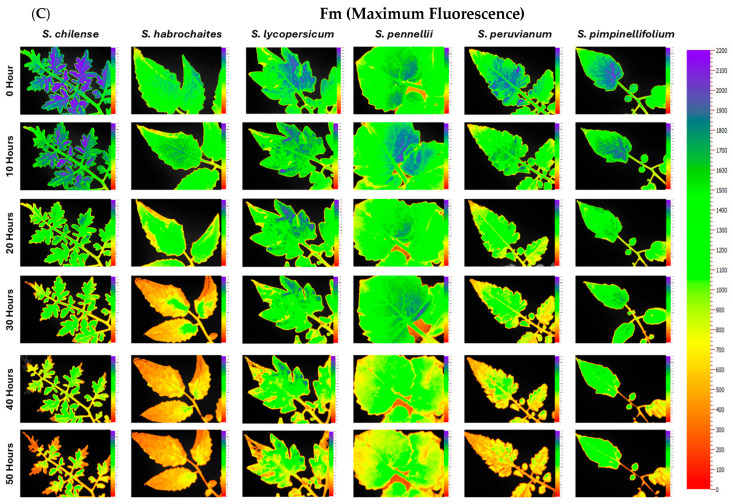
Chlorophyll *a* fluorescence (ChlaF) images of PSII in representative tomato leaf samples at 0, 10, 20, 30, 40, and 50 h after desiccation treatment. (**A**) Maximum quantum yield of PSII (QY_max), (**B**) minimal fluorescence (F0), (**C**) maximum fluorescence (Fm) in dark-adapted leaves. The color scale represents the quantitative variation in chlorophyll fluorescence parameters (QY_max, F0 and Fm), where blue/purple indicate higher fluorescence values, green represents intermediate values, and yellow to red indicate lower values.

**Figure 2:**
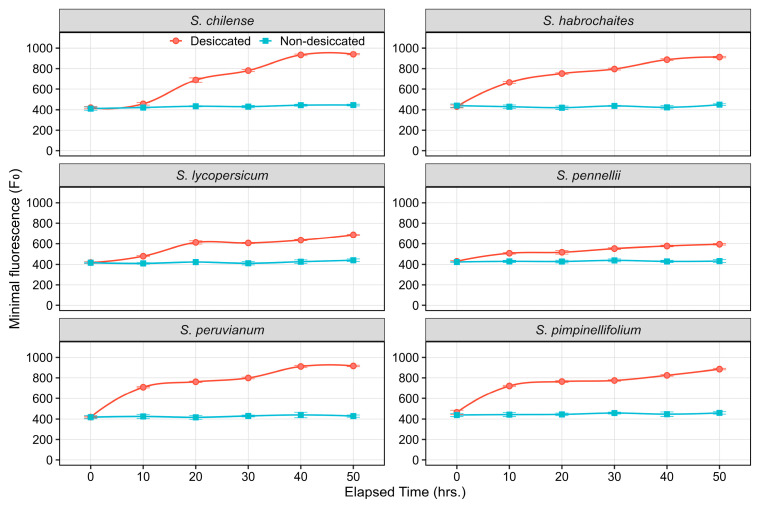
Line plots illustrating the temporal dynamics of F0 in non-desiccated and desiccated leaves of wild tomato species. Data points represent the mean of six replicates at each time point, with error bars indicating the standard error of the mean (SEm).

At the onset of desiccation (0 h), PSII efficiency is on par in all species; however, with the progression of desiccation up to 50 h, a significant interspecific difference is observed. The minimal chlorophyll fluorescence (F0) was monitored over 50 h to assess the impact of desiccation stress on the integrity of PSII across six *Solanum* species (Figure 1). For all species in the non-desiccated condition, F0 remained stable throughout the 50-h measurement period, maintaining a baseline value of approximately 400–450 units (Supplementary Figure S1). In contrast, desiccation stress induced a significant and progressive increase in F0 in every species examined, indicating damage to the light-harvesting complex or disruption of PSII reaction centers. The increase was generally observed within the first 10 h and continued until the 50-h time point. The magnitude of this increase varied among species; the accessions *S. chilense*, *S. habrochaites*, *S. peruvianum*, and *S. pimpinellifolium* exhibited the highest final F0 values, reaching approximately 900–1000 after 50 h of desiccation (Figure 2). Among all, *S. pennellii* showed a comparatively lower final maximal F0 value, stabilizing between 650 and 750 (Figure 2). Despite this quantitative difference, the qualitative response (progressive increase in F0) was consistent across all six species under desiccation stress.

**Figure 3:**
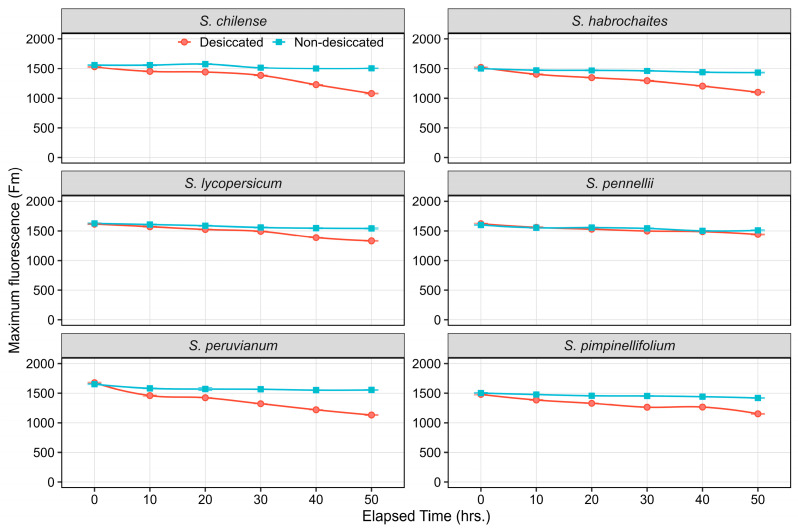
Line plots illustrating the temporal dynamics of Fm in non-desiccated and desiccated leaves of wild tomato species. Data points represent the mean of six replicates at each time point, with error bars indicating the standard error of the mean (SEm).

Maximum chlorophyll fluorescence (Fm) declined progressively in all species under desiccation as compared to non-desiccated control. Among wild relatives, *S. chilense*, *S. habrochaites*, and *S. peruvianum* showed the maximum decrease in Fm under desiccation, with values dropping from 1600–1700 at 0 h to 1100–1200 by 50 h, indicating substantial impairment of PSII reaction centers as desiccation progressed (Figure 3). *S. pimpinellifolium* exhibited a similar declining trend, though the reduction was slightly less pronounced than in *S. peruvianum* and *S. habrochaites*. In contrast, *S. pennellii* maintained comparatively stable Fm values under desiccation, with only a modest decrease (1600 to 1450), suggesting better stability of PSII photochemistry relative to other species (Figure 3). The cultivated species *S. lycopersicum* also showed a moderate decline in Fm, though the reduction was more pronounced than in *S. pennellii*, indicating comparatively lower tolerance to desiccation-induced stress.

**Figure 4:**
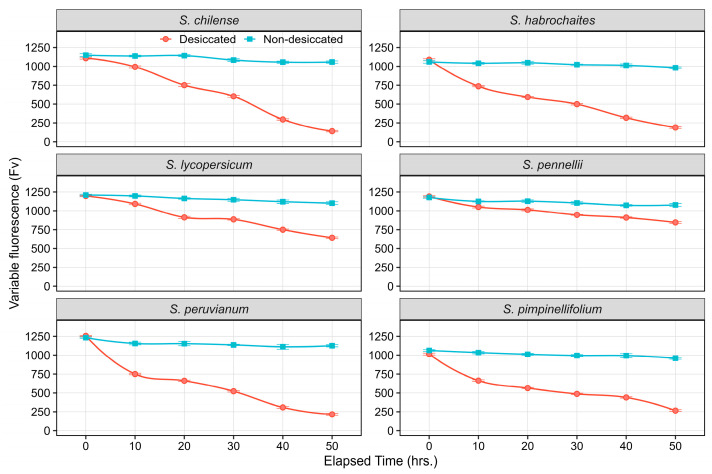
Line plots illustrating the temporal dynamics of Fv in non-desiccated and desiccated leaves of wild tomato species. Data points represent the mean of six replicates at each time point, with error bars indicating the standard error of the mean (SEm).

Variable fluorescence (Fv: Fm–F0), an indicator of the efficiency of PSII photochemical activity. Variable fluorescence declined abruptly across all species under desiccation, whereas non-desiccated controls maintained relatively stable values throughout the 50-h interval. Among wild relatives, *S. chilense*, *S. habrochaites*, *S. pimpinellifolium,* and *S. peruvianum* showed a significant decline in Fv, with values dropping from 1100–1250 at 0 h to <250 by 50 h, reflecting substantial loss of PSII photochemical capacity under severe desiccation. In contrast, *S. pennellii* displayed the highest retention of Fv under desiccation, and it decreased moderately (1150 to 850 units), indicating superior maintenance of PSII functionality compared to other species (Figure 4). The cultivated species *S. lycopersicum* showed an intermediate response, with Fv declining steadily to 700 units at 50 h, demonstrating moderate susceptibility compared with tolerant wild relatives.

**Figure 5:**
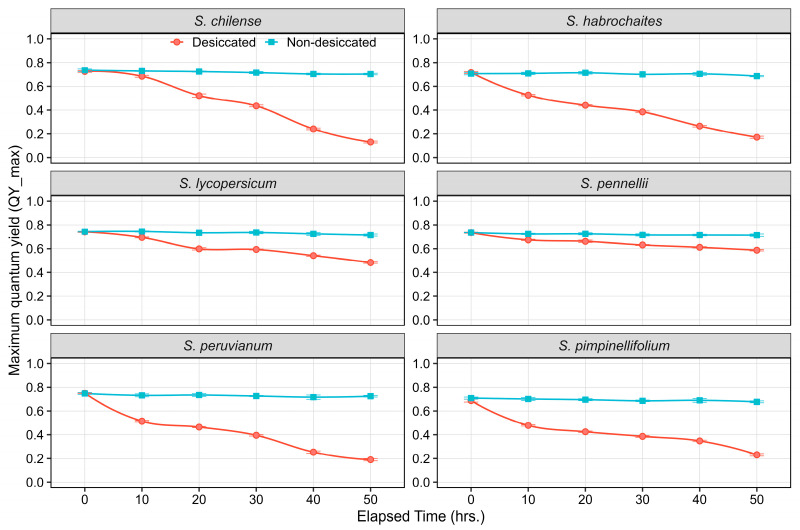
Line plots illustrating the temporal dynamics of QY_max in non-desiccated and desiccated leaves of wild tomato species. Data points represent the mean of six replicates at each time point, with error bars indicating the standard error of the mean (SEm).

Significant variation was observed in the maximum quantum yield of PSII (QY_max). The PSII declined significantly under desiccation across all tomato species, whereas non-desiccated controls maintained stable QY_max throughout the 50-h desiccation period. Among the wild species, *S. chilense*, *S. habrochaites*, *S. pimpinellifolium,* and *S. peruvianum* showed the steepest decline, with QY_max values decreasing from ~0.70–0.75 at 0 h to below 0.20 by 50 h, indicating severe impairment of PSII photochemical efficiency under desiccation treatment (Figure 5). In comparison, *S. lycopersicum* displayed a moderate reduction in QY_max, falling from 0.72 to 0.45 by 50 h, although it was sensitive to desiccation than highly tolerant species *S. pennellii* (Figure 5). Notably, *S. pennellii* maintained the highest QY_max under desiccation stress, with a gradual and minimal decline from 0.73 to 0.60, demonstrating superior PSII efficiency even at 50 h time point.

### 2.3. Functional Diversity of PSII Efficiency in Tomato Species Under Desiccation Stress

At the onset of treatment (0 h), all species exhibited similar PSII efficiency, but progressive desiccation induced marked interspecific variation. As desiccation progressed from 0 to 50 h, variation in PSII efficiency among the species became more pronounced, becoming significantly evident after 30 h of desiccation (Figure 5). Significant differences were observed in all measured ChlaF parameters (F0, Fm, Fv, and QY_max) in desiccated and non-desiccated treatments across the tomato species. Overall, desiccation stress caused a marked increase in minimal fluorescence (F0) and a concomitant decline in both maximum fluorescence (Fm) and variable fluorescence (Fv), resulting in substantial reductions in maximum quantum yield efficiency. QY_max was lowest in desiccated leaves of *S. chilense* (0.14), *S. habrochaites* (0.18), and *S. peruvianum* (0.19), whereas *S. pennellii* maintained the highest efficiency (0.59), followed by *S. lycopersicum* (0.44) (Table 2). Efficiency of PSII of *S. pennellii* was approximately 4.2, 3.2, 3.1, and 1.3-fold higher than *S. chilense, S. habrochaites*, *S. peruvianum*, and *S. lycopersicum,* respectively.

**Figure 6:**
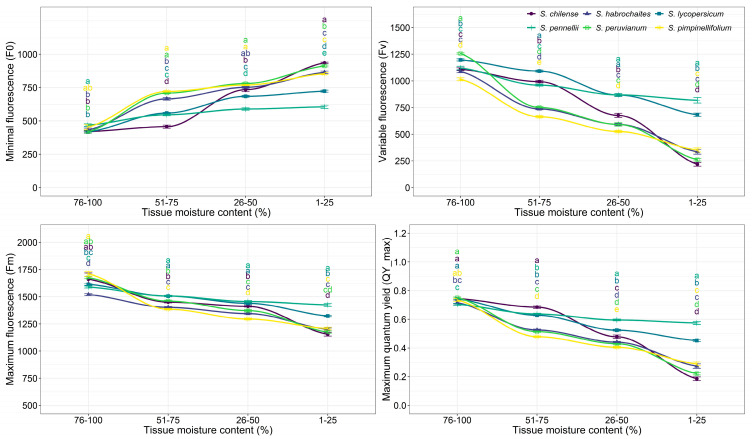
Response of chlorophyll *a* fluorescence parameters (QY_max, F0, Fv, and Fm) to changes in tissue moisture content (%) in leaves of tomato species. Mean values within each TMC group with identical letters indicate no significant difference, as determined by the LSD test.

Across all species, non-desiccated controls consistently showed high QY_max values (0.68–0.73), confirming that reductions in photochemical efficiency were influenced by desiccation stress. Both the main effects of species and desiccation treatment, as well as their interaction, were highly significant (*p* ≤ 0.001) for all ChlaF traits, demonstrating strong species-specific variation in PSII sensitivity to desiccation stress. Values of QY_max (Fv/Fm) below 0.70 indicate stress affected photo-inhibition of PSII. QY_max declined progressively with decreasing tissue moisture content (TMC) across all six tomato species (Figure 6). Under non-desiccated condition (76–100% TMC), QY_max values were comparable among species, ranging around 0.70–0.75, with no noticeable impairment of PSII efficiency. As TMC decreased to 51–75%, a substantial reduction in QY_max was observed. Although both *S. pennellii* and *S. lycopersicum* had higher QY_max, *S. pennellii* was statistically significant over *S. lycopersicum*. At moderate dehydration (26–50% TMC), interspecific differences became more pronounced. *S. pennellii* and *S. lycopersicum* retained relatively higher QY_max, whereas *S. chilense*, *S. habrochaites*, *S. peruvianum*, and *S. pimpinellifolium* exhibited a sharper decline (Figure 6). Further reduction in TMC to 1–25% resulted in a substantial decrease in QY_max across all species; however, *S. pennellii* exhibited significantly higher values than the remaining species. Under severe dehydration (1–25% TMC), QY_max dropped below 0.30 in *S. chilense*, *S. habrochaites*, *S. peruvianum*, and *S. pimpinellifolium* (Figure 6).

### 2.4. Retention of PSII Function

**Figure 7:**
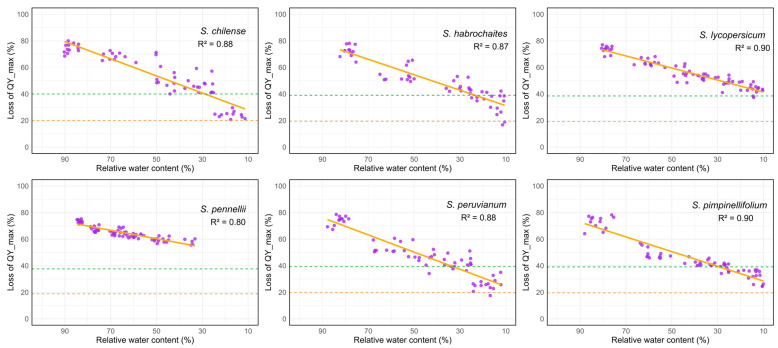
Effect of desiccation on maximum quantum yield of PSII (QY_max) as a function of declining leaf relative water content (RWC) in wild tomato species. Purple dots represent individual measurements across samples, while solid orange lines indicate fitted regression models. Dashed lines denote the thresholds corresponding to 50% (green) and 75% (orange) loss of the QY_max efficiency relative to initial values. Regression fits were significant (R^2^, *p* ≤ 0.01).

In all six tomato species studied, the photosynthetic II efficiency decreased as the leaf water content became lower. This decrease followed a strong and statistically significant linear pattern. The high R^2^ values (0.80 to 0.90) show that most of the variation in QY_max was explained by the reduction in leaf relative water content. Therefore, as desiccation progressed, the efficiency of PSII declined in a closely related and predictable way. The RWC corresponding to 50% loss of QY_max serves as a physiological threshold indicating the tolerance of PSII to dehydration stress (Figure 7). Species reaching this threshold even at higher RWC highlights their sensitivity, as substantial photochemical damage occurs earlier during water loss. *S. chilense*, *S. habrochaites*, *S. peruvianum*, and *S. pimpinellifolium* reached approximately 50% loss at relatively higher RWC levels (45–55%), reflecting earlier onset of PSII impairment during desiccation (Figure 7). In contrast, *S. lycopersicum* exhibited 50% QY_max loss at moderately lower RWC (40–45%). Particularly, *S. pennellii* showed the greatest resistance to dehydration-induced PSII damage, with 50% loss of QY_max occurring only at much lower RWC (30–35%).

**Figure 8:**
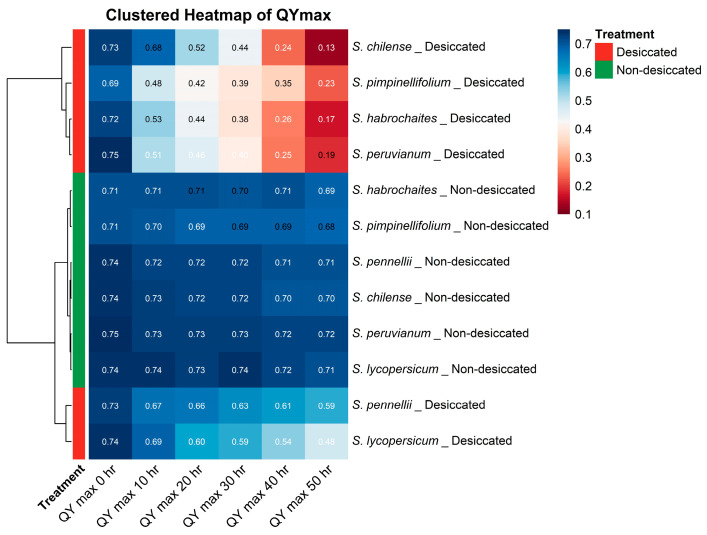
Clustered heatmap of maximum quantum yield of PSII (QY_max) in tomato species under desiccated and non-desiccated conditions across time points.

Additionally, hierarchical clustering-based heatmap analysis was performed across all species and time points under both desiccated and non-desiccated conditions to comprehensively visualize patterns of similarity and divergence in photosynthetic performance. Hierarchical clustering further revealed species-specific differences in PSII tolerance to desiccation. *Solanum pennellii* and *Solanum lycopersicum* were grouped in the same cluster due to their comparatively higher values under desiccated conditions, indicating greater stability of PSII efficiency during desiccation. In contrast, *S. chilense*, *S. habrochaites*, *S. peruvianum*, and *S. pimpinellifolium* formed a separate cluster characterized by markedly lower QY_max values, reflecting stronger impairment of PSII under severe desiccation. This approach enabled clear discrimination of genotypes based on their temporal responses and desiccation severity, and facilitated the identification of species exhibiting closer physiological behavior or contrasting tolerance levels (Figure 8).

## 3. Discussion

The present study provides a comprehensive evaluation of desiccation-induced physiological responses in cultivated and wild tomato species, with particular emphasis on tissue moisture dynamics and photosynthetic performance. By integrating measurements of tissue moisture content and chlorophyll *a* fluorescence parameters, the results offer interesting insights into the extent to which tomato species cope up with progressive leaf desiccation.

### 3.1. Effect of Tissue Moisture Content on ChlaF

Desiccation-induced loss in tissue moisture content (TMC) had a major impact on PSII functionality in both cultivated and wild tomato species, as observed by significant alterations in ChlaF parameters. The strong statistical influence of TMC on F0, Fm, Fv, and QY_max (*p* ≤ 0.001) underscores leaf hydration status as a key determinant of photosynthetic performance under dehydration stress. Similar moisture-dependent regulation of PSII efficiency has been widely reported across crop and wild species exposed to drought or desiccation stress [21,30,31].

The progressive increase in minimal fluorescence (F0) with declining TMC indicates impairment or inactivation of PSII reaction centers, likely caused by structural damage to thylakoid membranes and dissociation of light-harvesting complexes under dehydration. Elevated F0 values are a well-established indicator of stress-induced damage to PSII, reflecting reduced energy transfer efficiency from antenna pigments to reaction centers [32,33]. The sharp rise in F0 observed in the lowest TMC group (1–25%) suggests severe disruption of PSII integrity under extreme desiccation, consistent with earlier findings in tomato and other crops under drought stress [34,35]. In contrast, maximal fluorescence (Fm) and variable fluorescence (Fv) declined significantly with decreasing leaf TMC, indicating a reduction in the maximum efficiency of PSII photochemistry. The substantial decline in Fv under severe dehydration reflects reduced electron transport efficiency and diminished ability of PSII to utilize absorbed light for photochemical reactions. Our findings are supported by previous studies demonstrating that water deficit leads to conformational changes in PSII core proteins and reduced availability of Q_A_ (primary quinone electron acceptors), resulting in lowered Fv values [36,37]. The observed reduction in Fv under low TMC conditions thus confirms progressive photochemical inhibition as desiccation severity increases.

The sharp decline in QY_max at critically low TMC levels (1–25%) indicates that severe dehydration disrupts primary photosystem center, likely through damage to the D1 protein, dissociation of light-harvesting complexes, and accumulation of reactive oxygen species [38]. Thus, maintenance of leaf tissue moisture content between 50 and 75% range appears critical for preserving functional PSII efficiency in tomato, whereas prolonged exposure to TMC below 25% leads to near-complete loss of potential photosynthetic capacity. This threshold response highlights the sensitivity of PSII to dehydration stress and underscores the importance of tissue moisture status as a key determinant of drought tolerance in tomato species. The overall decline in QY_max with tissue desiccation reflects progressive damage or down regulation of PSII, a widely accepted indicator of stress severity and reduced potential photosynthetic capacity under drought [39,40]. A QY_max value ranged between 0.70 and 8.3 is typically associated with non-stressed, healthy leaves, whereas values below ~0.70 indicate photoinhibition and irreversible damage to PSII [17,41,42]. The drastic reduction in QY_max at low TMC suggests strong photo-inhibitory stress and loss of PSII repair capacity under dehydration, as reported previously in drought-sensitive tomato genotypes [22]. According to the model proposed by Guadagno et al. [43], water constitutes an essential structural element of the photosystems and directly affects chlorophyll dynamics, resulting in changes in energy partitioning and, consequently, chlorophyll fluorescence. Similarly, Rane et al. [18] also demonstrated that desiccation stress leads to a substantial reduction in photosynthetic efficiency in various fruit crops, attributed to impaired PSII functionality.

### 3.2. Effect of Desiccation Duration on ChlaF Across Species

The present study elucidates the temporal dynamics and interspecific variability in PSII efficiency under desiccation stress across six *Solanum* species, including the domesticated tomato (*S. lycopersicum*) and its wild relatives. The onset of desiccation rapidly induced reduction in photochemical efficiency within the first 10 h, as evidenced by rising F0 levels in all tested species. However, the magnitude of these effects differed among species, highlighting inherent interspecific variation in PSII stability. The wild species *S. chilense*, *S. habrochaites*, *S. peruvianum,* and *S. pimpinellifolium* exhibited a steep increase in F0 and a stronger reduction in Fm, indicating that their PSII units were more prone to damage under desiccating conditions. The comparatively lower F0 and moderate decline in Fm observed in *S. pennellii* suggest enhanced photoprotective capacity and higher structural stability of PSII under water deficit. Maintenance of lower baseline fluorescence in *S. pennellii* may reflect efficient energy dissipation mechanisms, such as elevated non-photochemical quenching and antioxidant-mediated protection against desiccation-induced oxidative stress [44,45].

Variable fluorescence (Fv) and maximum quantum efficiency of PSII further supported the existence of distinct tolerance phenotypes. All species displayed significant declines in Fv and QY_max with desiccation, confirming that PSII functionality deteriorates with declining tissue water content. Nonetheless, the rate and extent of photochemical impairment were highly species dependent. *S. pennellii* exhibited remarkable stability, retaining more than 80% of its initial Fv and maintaining QY_max above 0.6 after 50 h of desiccation, whereas other wild species showed QY_max values below 0.2, a range typically associated with photoinhibition and irreversible PSII damage [46]. This enhanced stability of *S. pennellii* may be linked to superior antioxidant capacity, accumulation of compatible solutes, or more effective non-photochemical quenching that limits excess excitation energy during dehydration [47]. The cultivated *S. lycopersicum* displayed an intermediate response, suggesting partial retention of PSII function but vulnerability beyond moderate dehydration levels. This observation is consistent with reports that desiccation-tolerant species possess enhanced membrane stability, protective pigments, and efficient photoprotective mechanisms that restrict irreversible PSII damage [48,49].

The significant interaction between species and stress further confirmed species-specific variations in PSII performance. The consistent high QY_max in non-desiccated treatment (0.70–0.75) across all species validates that decreased photochemical efficiency was a direct consequence of desiccation rather than genetic background per se. The findings thus highlight *S. pennellii* as an exceptional PSII efficiency under desiccation, whereas *S. chilense* and *S. habrochaites* exhibited high photosynthetic sensitivity. Plants subjected to a water deficit reduce transpiration by closing their stomata. Cuticular waxes form a protective barrier that minimizes non-stomatal transpiration over the entire leaf surface, thereby reducing passive water loss. Studies indicate that the effectiveness of this barrier depends more on wax composition and structural organization than on cuticle thickness or total wax quantity [50]. Among wild tomato relatives, *Solanum pennellii* exhibits abundant type IV trichomes, with densities frequently exceeding 70 trichomes mm^−2^, highlighting its value as a rich genetic resource for trichome-associated traits [51]. Greater cuticular wax deposition coupled with higher trichome density supports the observed desiccation tolerance in *S. pennellii*, likely by reducing transpirational water loss and enhancing protection against dehydration stress. These interspecific differences emphasize the potential of exploiting wild *Solanum* germplasm, particularly *S. pennellii*, as a donor of stress-resilient traits in tomato breeding programs targeting drought or dehydration tolerance.

### 3.3. Retention of PSII Function Under Progressive Dehydration

The significant negative linear relationship observed between relative water content and maximum quantum yield of PSII (QY_max) across *Solanum* species highlights the profound sensitivity of photosystem II to water availability. The high coefficients of determination (R^2^ = 0.80–0.90) indicate that reductions in QY_max were largely governed by water deficit, confirming that dehydration is a major factor for loss of photochemical efficiency. This finding aligns with previous reports describing that even moderate declines in cellular hydration can trigger structural and functional perturbations in the PSII complex, including detachment of the oxygen-evolving complex, reduced electron transport, and impaired D1 protein turnover [52,53].

The RWC value at which PSII efficiency declined by 50% serves as a meaningful physiological indicator for evaluating desiccation tolerance among species. In chlorophyll fluorescence studies, a 50% reduction in PSII photochemical efficiency is often considered a critical point at which substantial impairment of PSII function occurs, indicating the transition from moderate stress to severe photochemical limitation [21]. In the current study, species-specific thresholds delineated distinct tolerance strategies. The wild species *S. chilense*, *S. habrochaites*, *S. peruvianum*, and *S. pimpinellifolium* exhibited early onset of PSII impairment, reaching 50% QY_max loss at relatively high RWC levels (45–55%). This suggests that their PSII systems are unable to maintain photochemical stability once cellular hydration drops below moderate levels, consistent with rapid membrane destabilization and decline in thylakoid functionality often observed in sensitive genotypes [54,55]. Conversely, *S. pennellii* showed markedly greater resistance to dehydration-induced PSII damage, with QY_max declining by 50% only when relative water content dropped to 30–35%. These findings reinforce earlier physiological and molecular studies identifying *S. pennellii* as a valuable donor of drought-tolerance traits, including traits related directly to PSII stability and photoprotective capacity [29,56].

We could find that among the tomato wild species *S. pennellii* has superior PSII efficiency at low tissue moisture content. However, future research should integrate multi-omics approaches to elucidate the molecular underpinnings of observed differences, such as gene expression in PSII subunits or antioxidant pathways. Since this study employed a detached leaf assay to assess photosynthetic efficiency, the findings are limited to leaf-level responses. In addition, leaf samples were selected based on visual assessment, which may introduce some degree of subjectivity; however, standardized criteria such as comparable developmental stage, leaf size, and absence of visible damage were followed to maintain sample uniformity. Future studies incorporating whole-plant physiological measurements, such as gas exchange analysis, would provide a more comprehensive understanding of desiccation tolerance in these species.

Notably, this study provides the insights supporting the application of chlorophyll *a* fluorescence for assessing desiccation tolerance in wild tomato germplasm. The integration of imaging-based ChlaF emerges as a robust and novel imaging approach, enabling the resolution of both temporal and spatial heterogeneity in PSII efficiency within crop wild relatives. Moreover, these findings underscore the utility of chlorophyll *a* fluorescence parameters as reliable indicators of desiccation tolerance and PSII functional stability. The superior performance of *S. pennellii* highlights its potential as a valuable genetic resource for introgression of drought and desiccation resilience traits in cultivated tomato varieties potentially through breeding or biotechnological approaches.

## 4. Materials and Methods

### 4.1. Growth Conditions and Species Description

Tomato seeds were germinated in pro-trays containing a substrate composed of cocopeat, perlite, and vermiculite mixed in a 3:1:1 (*v*/*v*/*v*) ratio. One-month-old seedlings were subsequently transplanted into pots filled with soil. All pots were maintained in a greenhouse under controlled environmental conditions throughout the experimental period at ICAR-National Institute of Abiotic Stress Management, located in Baramati, Pune district, Maharashtra, India. The greenhouse temperature was maintained at 30 ± 2 °C during the day and 20 ± 2 °C at night to ensure optimal growth conditions. Relative humidity was regulated between 60 and 70%, and photosynthetically active radiation (PAR) was maintained within 450–750 µmol m^−2^ s^−1^. Tomato seedlings were transplanted into 8-inch diameter plastic pots containing 4.4 kg of clay loam soil. The soil composition consisted of 67% clay, 20% silt, and 13% sand, with a pH of 8.4, electrical conductivity of 0.5 dS m^−1^, organic carbon of 0.8%, and available nutrients of 85 mg kg^−1^ nitrogen, 7.85 mg kg^−1^ phosphorus, and 70 mg kg^−1^ potassium. The details of the tomato and its wild relatives used in the present study are given in Table 3.

### 4.2. Collection of Leaf Samples

The third and fourth fully opened leaves from the top of six different o plants were collected. These leaves, along with their petioles, were harvested at the morning time from 8 am to 9 am and immediately placed in insulated containers with ice packs and kept in the dark. After a period of dark adaptation, only firm and healthy leaves were selected for further desiccation treatments.

### 4.3. Measurements of Chlorophyll Fluorescence (ChlaF)

**Figure 9:**
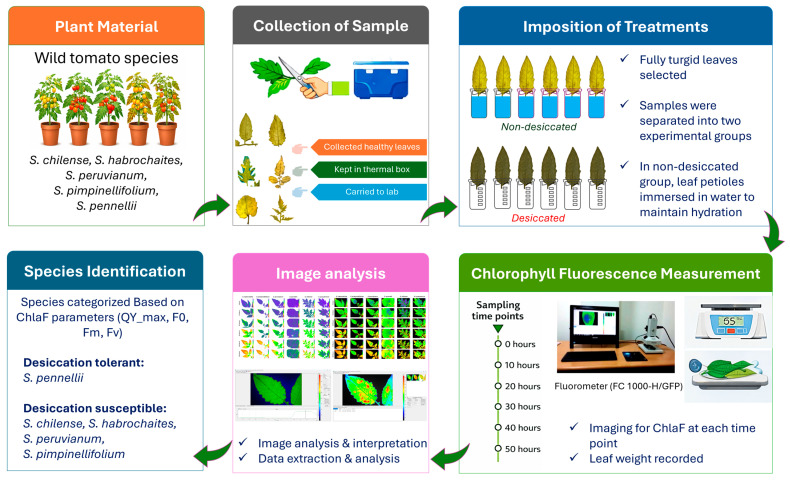
Schematic diagram illustrating the overview of experimental workflow.

To assess chlorophyll fluorescence parameters, 12 healthy leaves exhibiting no signs of senescence were selected. The leaves used in the experiment were fully expanded, visually healthy, and free from visible symptoms such as yellowing, chlorosis, necrotic spots, mechanical damage, or loss of leaf turgor. The leaves exhibiting any visible discoloration, marginal drying, or structural damage were excluded during sampling. These leaves were categorized into two treatment groups: non-desiccated and desiccated (Figure 9). Every group contained six leaves; each leaf represents a replicate. Then, samples were divided into two sets, in the first set of samples leaves were kept for desiccation treatment, while the second set was kept and maintained in distilled water to prevent dehydration through the experimental phase. Initial measurements were taken immediately after dark adaptation phase without any stress and considered that time point as 0 h for treatment groups, each containing different species. Following this, the petioles of the leaves in non-desiccated set were submerged in distilled water, ensuring no contact with the leaf lamina. All samples were kept at room temperature (27 °C) with a photon concentration of 170 μmol m^−2^ s^−1^ during the experiment. It took nearly 110–130 min to complete one set of ChlaF imaging measurements. Further, ChlaF was monitored at 0, 10, 20, 30, 40, and 50-h time intervals.

ChlaF in leaves was measured at different time intervals using an image fluorometer (FC 1000-H/GFP, Handy Fluor Cam, PSI, Brno, Czech Republic), as described by Nedbal et al. [58]. A high-sensitivity charge-coupled device camera (Fluor Cam 7) was used to detect fluorescence using the Fluor Cam software (version 7). First, images of the dark-adapted fluorescence level (F0) were taken using non-actinic measurement flashes that were powered by super-bright LEDs. To measure the maximum fluorescence (Fm) level, a saturating pulse of light radiation (2500 μmol photons m^−2^ s^−1^) with a duration of 800 milliseconds was applied. The maximum photochemical efficiency of PSII (QY_max) was calculated as follows.

$$\frac{\mathrm{Fv}}{\mathrm{Fm}}=\frac{\mathrm{Fm}-F0}{\mathrm{Fm}}$$

Fv: variable fluorescence, F0: minimal fluorescence, Fm: maximum fluorescence.

Pixel values for Fv/Fm were visually represented using a false color scale, where red indicates 0.00 and blue represents 0.80. The difference between the maximum (Fm) and minimum (F0) fluorescence yields is the variable fluorescence (Fv). The desiccation response of different tomato species was determined using the ratio of the Fv/Fm of individual leaves under both dehydration and hydration conditions to determine the percent loss of the maximum quantum yield of photosystem II, which is an indicator of reduced fluorescence ability during desiccation treatment. Conventional physiological measurements of photosynthetic efficiency frequently involve bulk analyses, which are typically more time consuming and labor intensive compared with fluorescence-based techniques. Chlorophyll *a* fluorescence imaging is a rapid, reliable technique that enables the assessment of photosystem II (PSII) performance.

### 4.4. Measurement of Relative Water Content and Leaf Tissue Moisture Content

The tissue moisture content (TMC) of leaves was continuously monitored throughout the experiment. Fresh leaf mass (LM) was recorded at regular time points immediately before chlorophyll *a* fluorescence (ChlaF) imaging using a precision balance (BSA423S-CW, Sartorius, Göttingen, Germany). Each species with six replicates per treatment were recorded under both desiccated and non-desiccated condition. To determine the final dry weight of the leaves, they were dried in an oven at 65 °C for 72 h after the last measurement. The moisture content of the leaf tissue was calculated using the formula provided by Khapte et al. [21].

$$\mathrm{TMC}\;(\%)=\frac{\mathrm{LM}\;\mathrm{at}\;\mathrm{the}\;\mathrm{time}\;\mathrm{of}\;\mathrm{measurement}-\mathrm{Final}\;\mathrm{dry}\;\mathrm{mass}}{\mathrm{LM}\;\mathrm{at}\;\mathrm{the}\;\mathrm{beginning}\;\mathrm{of}\;\mathrm{the}\;\mathrm{experiment}\;}\;\times 100$$

The experiment was repeated two times, with each treatment including six replicates, to verify the reliability and reproducibility of the results. The relative water content (RWC) of the dehydrated leaves was analyzed using a modified version of the formula described by Khapte et al. [21].

$$\mathrm{RWC}\;(\%)=\frac{\mathrm{LM}\;\mathrm{of}\;\mathrm{dehydrated}\;\mathrm{leaf}-\mathrm{LM}\;\mathrm{of}\;\mathrm{dry}\;\mathrm{leaf}}{\mathrm{LM}\;\mathrm{at}\;\mathrm{the}\;\mathrm{beginning}\;\mathrm{of}\;\mathrm{the}\;\mathrm{experiment}-\mathrm{LM}\;\mathrm{of}\;\mathrm{dry}\;\mathrm{leaf}}\;\times \;100$$

### 4.5. Statistical Analysis

The experimental data was analyzed using R software (version 4.3.2; R Core Team, Vienna, Austria). The data normality was verified first using the Shapiro–Wilk test. Analysis of Variance (ANOVA) was conducted for the factorial Completely Randomized Design (CRD) using the ‘Doebioresearch’ and ‘agricolae’ packages. Mean comparisons were performed with the Least Significant Difference (LSD) test, applying a significance level of *p* ≤ 0.01. Moreover, to visualize and compare the trends of change in Chlorophyll *a* Fluorescence (ChlaF) parameters over time and across tissue moisture levels, a line curve was generated using the ‘geome smooth’ function in the ‘ggplot2’ package. The data points on this curve represent the means of the replicated treatments.

## 5. Conclusions

Among the chlorophyll *a* fluorescence parameters monitored during progressive leaf desiccation, QY_max emerged as one of the promising indicators for detecting changes in photosystem II (PSII) performance in tomato and its wild relatives. The results revealed interspecific variation in PSII responses to desiccation, with *Solanum pennellii* maintaining comparatively higher QY_max values under severe dehydration, suggesting greater stability of PSII photochemical efficiency relative to the other species examined. These findings highlight the utility of chlorophyll *a* fluorescence imaging as a tool to monitor temporal changes in photosynthetic performance during desiccation stress. The approach enabled the detection of differences in PSII activity among cultivated tomato and related wild species, providing insights into their physiological responses to desiccation. Overall, this study demonstrates the applicability of chlorophyll fluorescence imaging for evaluating desiccation-induced changes in PSII function in tomato germplasm and related species.

## Figures and Tables

**Table 1:** Influence of leaf tissue moisture content on chlorophyll *a* fluorescence parameters in tomato species.

TMC (%)	F0	Fm	Fv	QY_max
76–100	432.68 a	1541.38 a	1099.91 a	0.72
51–75	585.61 b	1470.64 b	902.42 b	0.60
26–50	727.97 c	1385.46 c	684.70 c	0.47
1–25	857.03 d	1211.94 d	366.41 d	0.28
*p* ≤ 0.001	***	***	***	***

QY_max: maximum quantum yield of PSII, Fv: variable fluorescence, Fm: maximum fluorescence, F0: minimal fluorescence, TMC: tissue moisture content. Based on the LSD test, mean values of the three replicates sharing the same letter within each column do not differ significantly at *p* ≤ 0.001. *** indicates significance at *p* ≤ 0.001.

**Table 2:** Mean ChlaF parameters of desiccated and non-desiccated leaves of wild tomato species at 50 h.

Treatment	F0	Fm	Fv	QY_max
*S. chilense*	692.15 a	1291.91 d	599.77 de	0.42 d
*S. habrochaites*	680.07 ab	1265.47 e	585.41 e	0.43 d
*S. lycopersicum*	590.69 c	1425.93 b	872.74 b	0.58 b
*S. pennellii*	513.38 d	1474.99 a	962.02 a	0.66 a
*S. peruvianum*	671.46 b	1342.31 c	670.85 c	0.46 c
*S. pimpinellifolium*	671.52 b	1284.6 d	613.09 d	0.46 c
Significance (S)	***	***	***	***
Desiccated	830.91 a	1201.69 b	383.42 b	0.3 b
Non-desiccated	442.18 b	1493.38 a	1051.21 a	0.71 a
Significance (D)	***	***	***	***
*S. chilense*:Desiccated	938.99 a	1080 f	141.02 i	0.14 h
*S. habrochaites*:Desiccated	911.06 b	1100 f	188.95 h	0.18 g
*S. lycopersicum*:Desiccated	741.51 d	1310 d	643.5 f	0.44 e
*S. pennellii*:Desiccated	594.9 e	1440 c	845.91 e	0.59 d
*S. peruvianum*:Desiccated	914.75 ab	1130.09 e	215.34 h	0.19 g
*S. pimpinellifolium*:Desiccated	884.24 c	1150 e	265.77 g	0.24 f
*S. chilense*:Non-desiccated	445.31 fg	1503.82 b	1058.52 c	0.71 ab
*S. habrochaites*:Non-desiccated	449.07 fg	1430.93 c	981.86 d	0.69 bc
*S. lycopersicum*:Non-desiccated	439.87 fg	1541.85 a	1101.98 ab	0.72 a
*S. pennellii*:Non-desiccated	431.86 g	1509.97 b	1078.12 bc	0.72 a
*S. peruvianum*:Non-desiccated	428.17 g	1554.53 a	1126.37 a	0.73 a
*S. pimpinellifolium*:Non-desiccated	458.81 f	1419.21 c	960.4 d	0.68 c
*Significance* (S x D)	***	***	***	***

Fm: maximum fluorescence, F0: minimal fluorescence, Fv: variable fluorescence, QY_max: maximum quantum yield. Mean values from three replicates with identical letters within each column indicate no significant difference as determined by the LSD test. *** denote significance at *p* ≤ 0.001.

**Table 3:** Description of tomato germplasm used in the study [57].

Species Name	Biological Status	Origin	Accession Number	Habitat/Climate	Breeding System	Source
*S. chilense*	Wild	Southern Peru, Northern Chile	EC-1092537	Hyper-arid desert, high radiation, cold nights	Self-incompatible	World Vegetable Center, Taiwan
*S. pimpinellifolium*	Wild	Coastal Ecuador and Peru	EC-3084438	Coastal dry tropics	Facultative Self-compatible	World Vegetable Center, Taiwan
*S. habrochaites*	Wild	Andes of Peru and Ecuador	EC-1092532	Mid–high altitude, humid montane	Self-incompatible	World Vegetable Center, Taiwan
*S. peruvianum*	Wild	Peru and Northern Chile	IIHR-2809	Arid and semi-arid valleys	Self-incompatible	ICAR-IIHR, Bangaluru
*S. pennellii*	Wild	Arid regions of Western Peru	EC-1092536	Desert scrub, low rainfall	Self-incompatible	World Vegetable Center, Taiwan
*S. lycopersicum*	Cultivated	Andean region; globally cultivated	85 (GT-3)	Cultivated environments	Self-compatible	ICAR-IIVR, Varanasi

## Data Availability

The datasets generated and/or analyzed during the current study are available from the corresponding author upon request.

## Supplementary Materials

The following supporting information can be downloaded at: https://www.mdpi.com/article/10.3390/plants15091339/s1, Figure S1: Chlorophyll *a* fluorescence (ChlaF) images of PSII in representative tomato leaf samples at 0, 10, 20, 30, 40, and 50-h under non-desiccated condition. (1A) Maximum quantum yield of PSII (QY_max), (1B) minimal fluorescence (F0), (1C) maximum fluorescence (Fm) in dark-adapted leaves. Table S1. Analysis of variance for ChlaF parameters in leaf tissue of tomato species.

## Abbreviations

The following abbreviations are used in this manuscript:

F0	Minimal fluorescence
Fm	Maximum fluorescence of dark-adapted leaves
Fv	Variable fluorescence
QY_max	Maximum quantum yield of PSII
PSII	Photosystem II
RWC	Relative water content
TMC	Tissue moisture content
ChlaF	Chlorophyll *a* Fluorescence
