# Supplementary material for: Chlorophyll Fluorescence-Based High-Throughput Phenotyping Reveals Mechanisms and Enables Rapid Screening of Desiccation-Tolerant Wild Tomato Species
Source: Plants (Basel). 2026 Apr 28;15(9):1339. doi: 10.3390/plants15091339 (PMC13165329; doi:10.3390/plants15091339)
Supplement: Supplementary file 1 [file plants-15-01339-s001.zip › plants-4266145-supplementary.pdf]

S1A

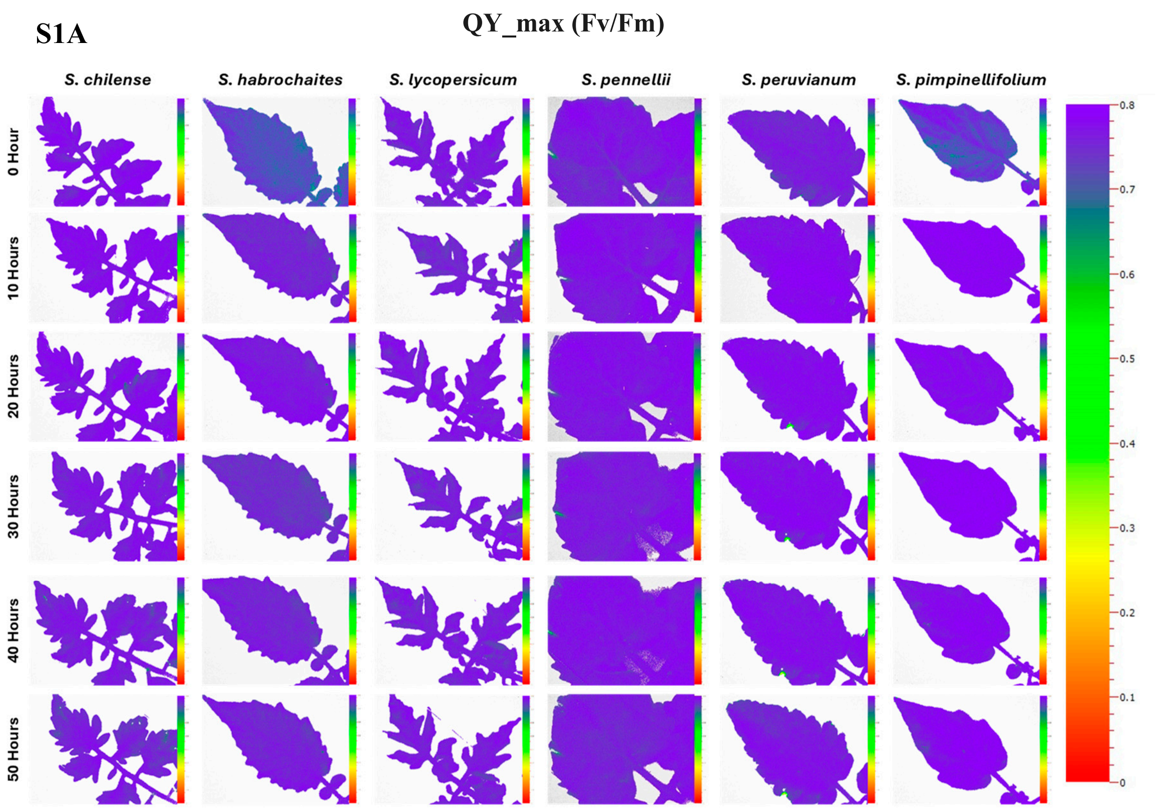
QY_max (Fv/Fm)
S. chilense
S. habrochaites
S. lycopersicum
S. pennellii
S. peruvianum
S. pimpinellifolium
0 Hour
10 Hours
20 Hours
30 Hours
40 Hours
50 Hours
0.8
0.7
0.6
0.5
0.4
0.3
0.2
0.1
0

S1B

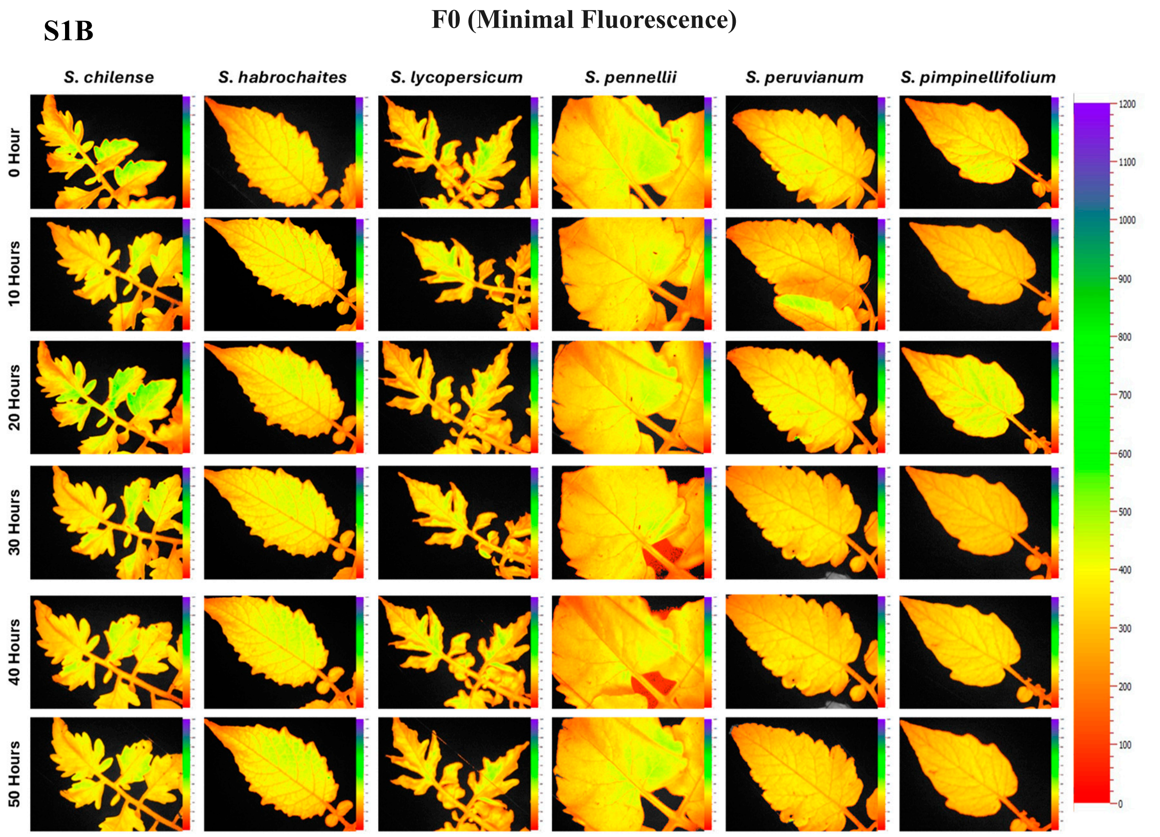
F0 (Minimal Fluorescence)
S. chilense
S. habrochaites
S. lycopersicum
S. pennellii
S. peruvianum
S. pimpinellifolium
0 Hour
10 Hours
20 Hours
30 Hours
40 Hours
50 Hours
1200
1100
1000
900
800
700
600
500
400
300
200
100
0

S1C Fm (Maximum Fluorescence)

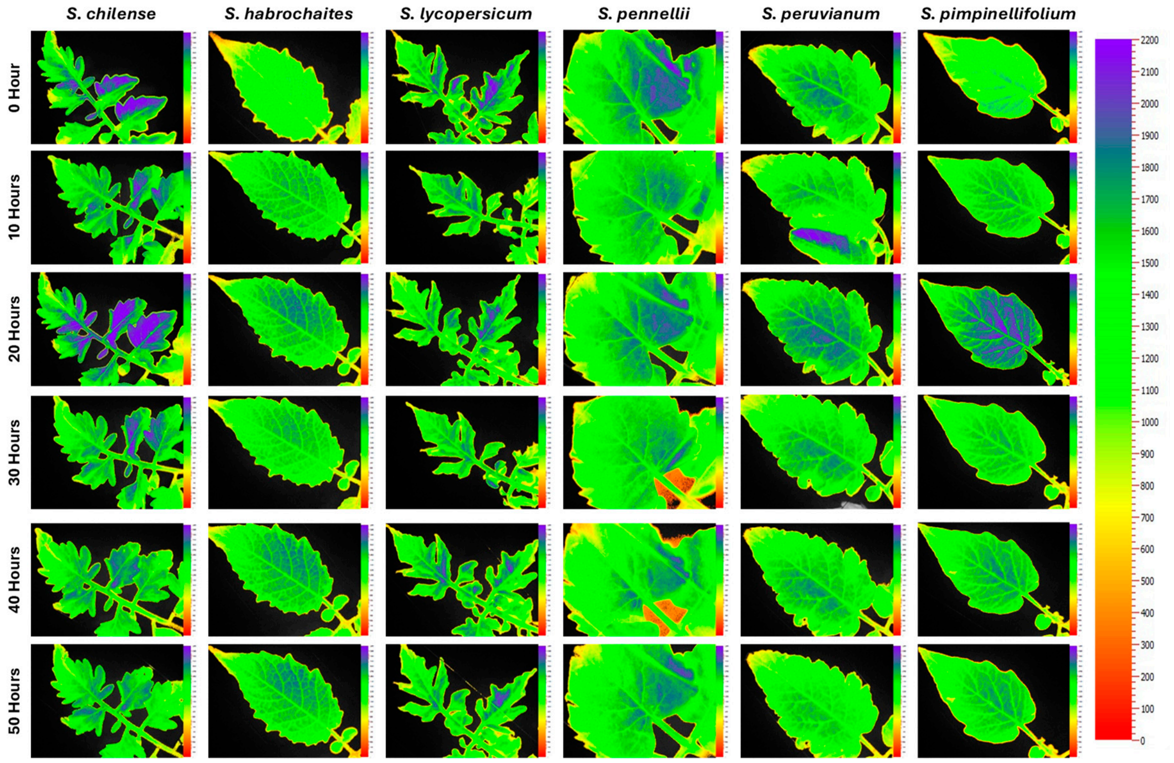

Supplementary Figure S1. Chlorophyll a fluorescence (ChlaF) images of PSII in representative tomato leaf samples at 0, 10, 20, 30, 40 and 50 hours under non-desiccated condition. (1A) Maximum quantum yield of PSII (QY_max), (1B) Minimal fluorescence (F0), (1C) Maximum fluorescence (Fm) in dark-adapted leaves. The colour scale represents the quantitative variation in chlorophyll fluorescence parameters (QY_max, F0 and Fm), where blue/purple indicate higher fluorescence values, green represents intermediate values, and yellow to red indicate lower values.

Supplementary Table S1. Analysis of variance for ChlaF parameters in leaf tissue of tomato species.

Source of variation	DF	F0	Fm	Fv	QY_max
Species	5	295462	491836	1844966	0.3711
TMC	5	7436027	3670857	19670001	6.9667
Species: TMC	25	96983	48926	290292	0.0994

F0: minimal fluorescence, Fm: maximum fluorescence, Fv: variable fluorescence, QY_max: maximum quantum efficiency of PSII, DF: degrees of freedom, TMC: tissue moisture content
